# RNaseIII and T4 Polynucleotide Kinase sequence biases and solutions during RNA-seq library construction

**DOI:** 10.1186/1745-6150-8-16

**Published:** 2013-07-04

**Authors:** Changhoon Lee, R Adron Harris, Jason K Wall, R Dayne Mayfield, Claus O Wilke

**Affiliations:** 1 Waggoner Center for Alcohol and Addiction Research, Section of Neurobiology and Institute for Cellular and Molecular Biology, The University of Texas at Austin, Austin, TX 78712, USA; 2 Center for Computational Biology and Bioinformatics, Section of Integrative Biology and Institute for Cellular and Molecular Biology, The University of Texas at Austin, Austin, TX 78712, USA

**Keywords:** RNaseIII, T4PNK, Sequence bias, Heat fragmentation, OptiKinase, RNA-seq

## Abstract

**Background:**

RNA-seq is a next generation sequencing method with a wide range of applications including single nucleotide polymorphism (SNP) detection, splice junction identification, and gene expression level measurement. However, the RNA-seq sequence data can be biased during library constructions resulting in incorrect data for SNP, splice junction, and gene expression studies. Here, we developed new library preparation methods to limit such biases.

**Results:**

A whole transcriptome library prepared for the SOLiD system displayed numerous read duplications (pile-ups) and gaps in known exons. The pile-ups and gaps of the whole transcriptome library caused a loss of SNP and splice junction information and reduced the quality of gene expression results. Further, we found clear sequence biases for both 5' and 3' end reads in the whole transcriptome library. To remove this bias, RNaseIII fragmentation was replaced with heat fragmentation. For adaptor ligation, T4 Polynucleotide Kinase (T4PNK) was used following heat fragmentation. However, its kinase and phosphatase activities introduced additional sequence biases. To minimize them, we used OptiKinase before T4PNK. Our study further revealed the specific target sequences of RNaseIII and T4PNK.

**Conclusions:**

Our results suggest that the heat fragmentation removed the RNaseIII sequence bias and significantly reduced the pile-ups and gaps. OptiKinase minimized the T4PNK sequence biases and removed most of the remaining pile-ups and gaps, thus maximizing the quality of RNA-seq data.

**Reviewers:**

This article was reviewed by Dr. A. Kolodziejczyk (nominated by Dr. Sarah Teichmann), Dr. Eugene Koonin, and Dr. Christoph Adami. For the full reviews, see the Reviewers' Comments section.

## Background

Next generation sequencing is revolutionizing biological data acquisition. It can be used instead of many existing specialized measurement approaches. For example, one of the next generation sequencing techniques, RNA-seq, has replaced microarrays in nearly all application areas. Microarrays detect gene expression levels based on known probes. By contrast, RNA-seq does not have this limitation. It can measure expression levels in any genomic region. In addition, it also provides information about SNPs and the location of splicing junctions [1-3].

However, one disadvantage of RNA-seq is that its sequence data can be biased by library construction. For example, mapped read counts can over or underestimate true RNA abundances based on a variety of library construction steps, such as reverse transcription, adaptor ligation, or amplification [4-7]. In a comparison of multiple library preparation methods for RNA-seq, all methods introduced their own bias and showed different expression patterns from the same sample [8]. Here, we developed three new RNA-seq library preparation methods and compared them with the current RNA-seq library construction method for the SOLiD system. We identified the step in library preparation that caused the most pronounced bias and outlined alternative preparation techniques that can virtually eliminate this concern.

## Results

### RNA-seq library construction

RNA-seq library construction methods vary among sequencing instruments. ABI SOLiD, Illumina HiSeq, and Roche 454 are three major RNA-seq sequencers. They have their own library preparation methods, and other companies also developed library construction methods for the sequencers. Furthermore, customized methods have been designed based on researchers’ interests, for example, RNA-editing or poly(A) tail studies [9,10]. In previous studies, random primers or oligo(dT) primers were used for reverse transcription of whole transcriptome library preparation [5,10,11].

**Figure 1:**
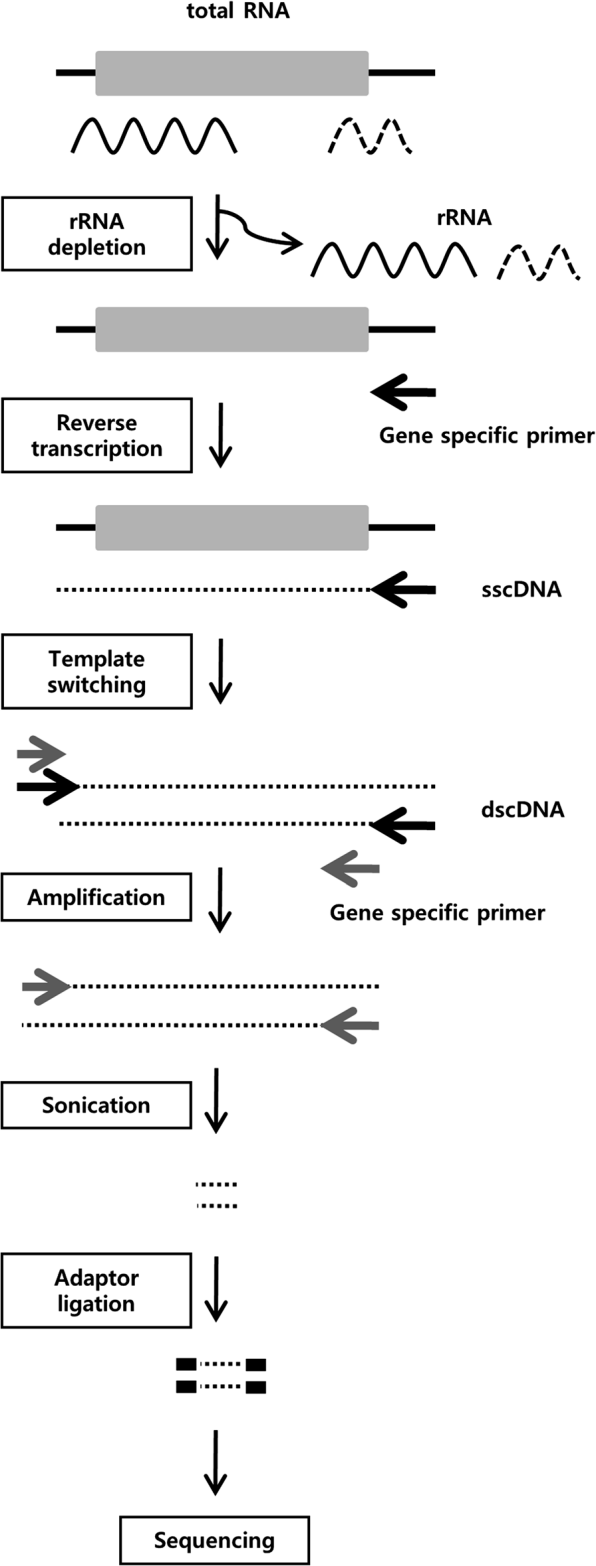
**Preparation of gene specific library.** mRNA is visualized as a grey box flanked by black lines. The grey box represents the open reading frame (ORF), and the black lines represent UTRs. We obtained total RNA and depleted rRNA from it. Reverse transcription was carried out using a GABAB1 gene specific primer. The resulting single stranded complementary DNAs (sscDNAs) were converted to dscDNAs via template switching. After amplification with another gene specific primer, dscDNAs were sonicated. Adaptor ligated dscDNAs were sequenced with the SOLiD system.

In this study, we compared different whole transcriptome libraries to each other as well as to a library specific to the human GABAB1 gene. There was substantial difference between the two library preparations. For the gene specific library, complementary DNA (cDNA) was fragmented after gene specific reverse transcription and amplification, and adaptors were ligated to the fragmented cDNA (Figure 1). For the gene specific reverse transcription and amplification, the gene specific primers were designed for the 3′ untranslated region (UTR) since most human GABAB1 transcripts share a common 3’ UTR [12]. To prepare the whole transcriptome library, RNA was fragmented first, and adaptors were ligated to the end of the fragmented RNA. The RNA was reverse transcribed based on the random sequence overhangs of the adaptors. Though this approach generated cDNAs based on random sequences, it can be considered targeted priming because the reverse transcription started from the end of the fragmented RNA. The ends of the cDNA always reflected the 5′ and 3′ end sequences of the fragmented RNA. For our study, the gene specific and whole transcriptome libraries were prepared for the SOLiD system and sequenced.

### Sequence mapping and visualization

**Figure 2:**
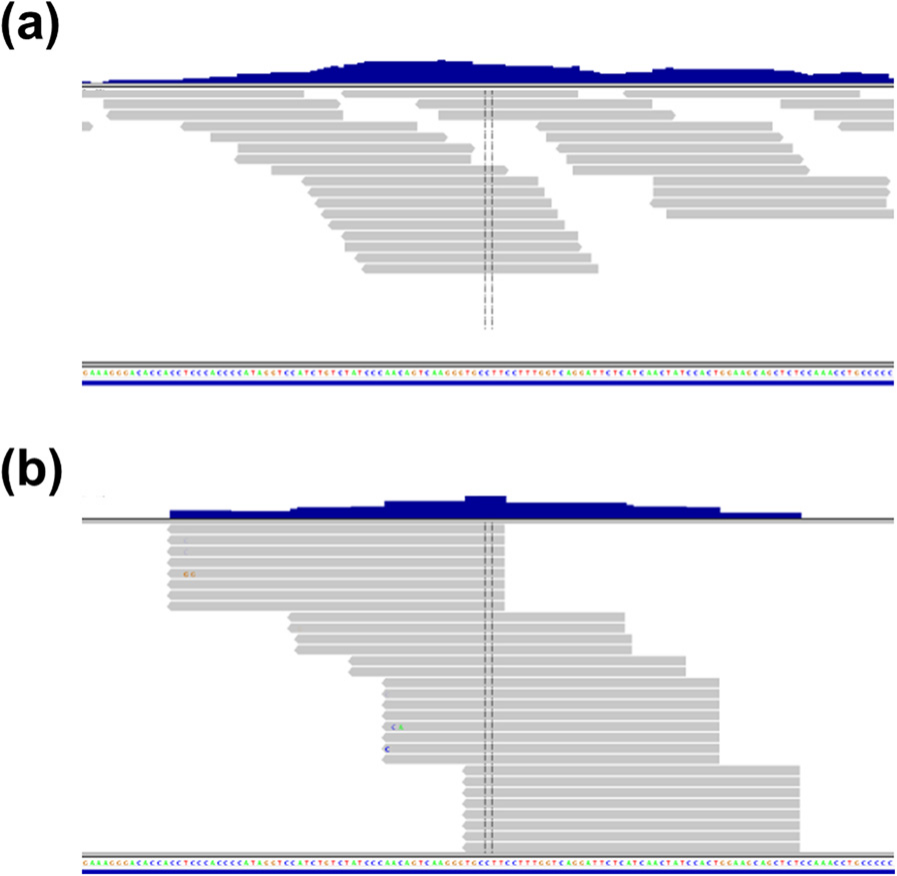
**Mapping patterns of the gene specific library and the whole transcriptome library. (a)** Mapped reads for the gene specific library were visualized at GABAB1 using IGV. **(b)** For the same genomic region as in (a), mapped reads for the whole transcriptome library were visualized. Compared to the gene specific library, the whole transcriptome library showed read pile-ups as well as gaps. At the gaps, no read was mapped at a known exon even though the gene specific library reads were mapped to these sites. In each figure, the top blue part shows the coverage of mapped reads, the middle grey part shows for individual mapped reads, and the bottom shows chromosome sequences and gene structures.

Raw sequence reads of the gene specific and whole transcriptome libraries were filtered by sequence quality values. Non-coding RNAs and adaptor sequences were also removed. The non-coding RNAs were primarily ribosomal RNAs (rRNAs) and transfer RNAs (tRNAs). After filtering, the sequence data of the gene specific and whole transcriptome libraries were mapped against human reference genome, hg18, and visualized at the GABAB1 gene (Figure 2).

For each step, the remaining read numbers were calculated (Additional file 1: Table S1). Overall mappability of these reads was 29.2%. Redundancy (redundant mapped reads / mapped reads) was also calculated for each library. The number of mapped reads and the redundancy have a strong positive correlation within the same libraries. Because more mapped reads can have more chances to be identical to other reads, the libraries that generated more mapped reads showed higher redundancies. Different library construction methods also resulted in different redundancies even though they had the same amplification cycles and generated similar mapped reads.

Figure 2a shows the mapped reads that resulted from the gene specific library for the GABAB1 gene. Figure 2b illustrates the mapped reads resulting from the whole transcriptome library for the same chromosome location. These reads were 50 mer single-end reads. We found the mapping patterns between these two libraries to be dramatically different. Even though the chromosome region was a known exon and should be covered with continuous reads, the mapped reads of the whole transcriptome library showed gaps and pile-ups. (We use the term *pile-up* to refer to a substantial number of reads having exactly the same sequence, as seen in Figure 2b). This uneven coverage of exons with mapped reads demonstrates that the whole transcriptome library has substantial bias. The pile-ups and gaps of the whole transcriptome library were ubiquitously found throughout the genome, including for endogenous housekeeping genes, such as ACTB, PPIA, GAPDH, and PGK1 (Additional file 2: Figure S1). Therefore, we concluded that the pile-up and gap pattern was not a mapping artifact of a specific gene.

### Comparison of the two sequencing data sets

**Figure 3:**
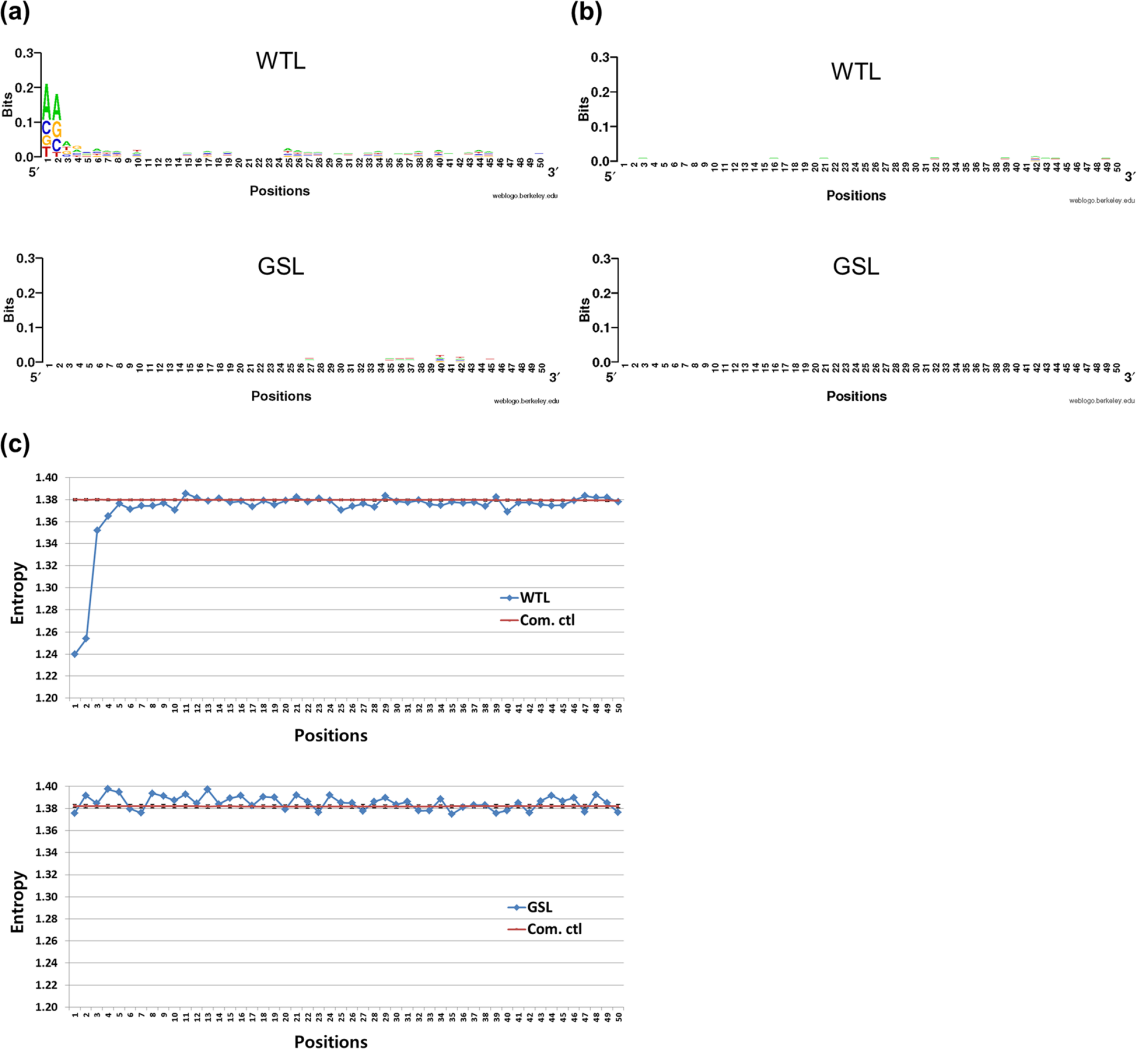
**Sequence logo and entropy analysis of mapped reads.** We analyzed mapped reads for deviations from randomness using sequence logo and entropy. (Note that the height of letters in the sequence logo is given by the reduction in entropy from random expectation. Thus, large letters in the sequence logo correspond to depressions in the entropy numbers.) **(a)** shows sequence logos for the whole transcriptome library (WTL) and gene specific library (GSL). **(b)** shows sequence logos for their computational controls (Com. ctl). **(c)** shows entropy data for whole transcriptome library and gene specific library including their computational controls. The entropies of computational controls have black error bars from 1000 replicates. The error bars represent their standard deviation, and they are smaller than the size of the symbols of whole transcriptome and gene specific libraries on the graph. The computational controls obtained by randomly generated sequences from human exome for each library. Based on mapped read locations and splicing junction information, we predicted existing 200 bp cDNA sequences in RNA-seq library. 50 bp partial sequences were randomly selected from the sequences. In all cases, we analyzed a window of 50 bp starting with the 5’ ends of the sequence fragments. The gene specific library showed almost no deviations from randomness. By contrast, the whole transcriptome library had a strong bias at the first 2 bases at the 5’ end.

The pile-ups shown in Figure 2b might be the result of sequence bias at the 5' end of the read. Fragment end sequence bias can generate read duplication under SOLiD sequencing and cause the pile-ups. To examine this possibility, we collected all mapped reads and calculated sequence logos (using WebLogo) and entropy at each nucleotide position (Figure 3). The height of letters in the sequence logo is calculated based on entropy at that site. Thus, entropy profiles and sequence logo profiles should be similar, except that entropy profiles were calculated on all reads and sequence logos were calculated on a random subset (see Methods). We found that the 5′ ends of reads from the whole transcriptome library have a specific sequence pattern. The sequence logo results showed a predominant pattern of AA at the 5′ end of the whole transcriptome library reads (Figure 3a). In contrast, reads from the gene specific library did not display this sequence pattern. For controls of each library, we generated random exome sequences *in silico* based on mapped read locations. These controls showed almost no bias whatsoever (Figure 3b). The strong bias in the whole transcriptome library was reflected in reduced entropy at the 5′ end of reads (Figure 3c). In contrast, entropy for the gene specific library showed only a minor fluctuation without the 5′ end drop.

### Comparison of the two library preparation methods

**Figure 4:**
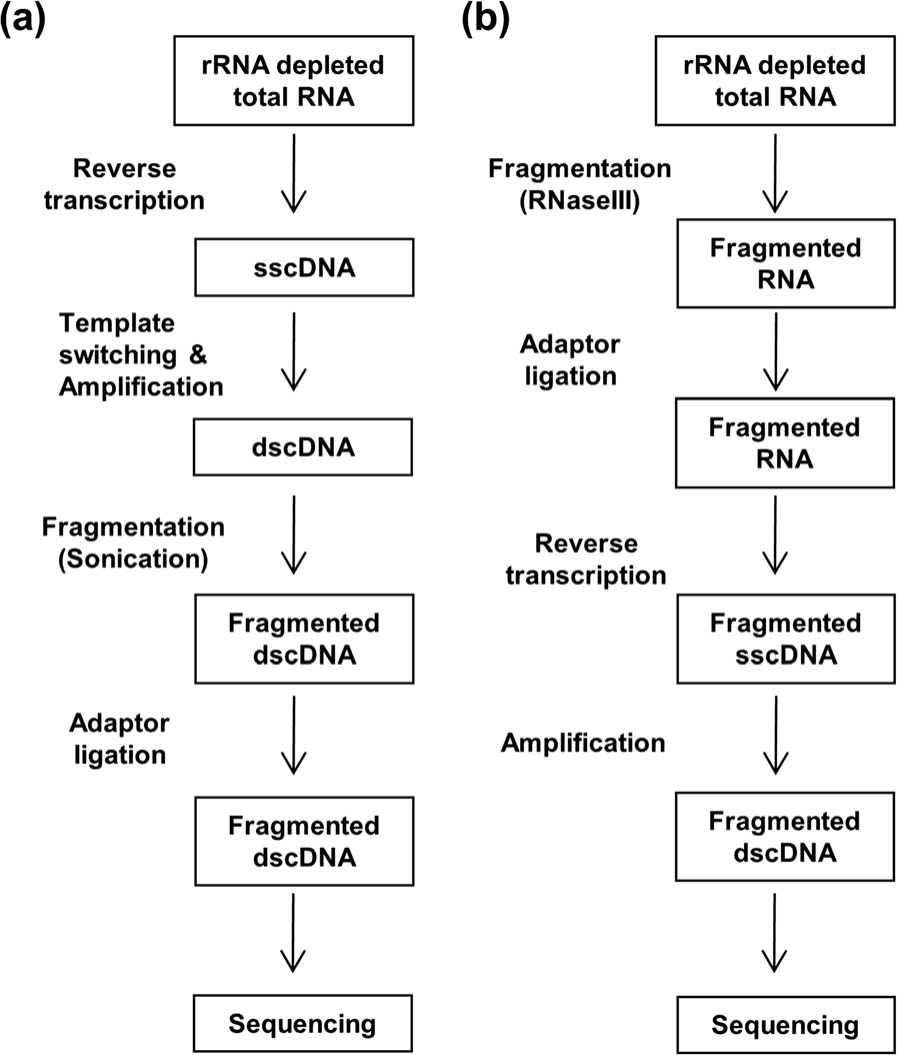
**Simplified construction methods of the gene specific library and the whole transcriptome library. (a)** The construction method of the gene specific library was simplified from Figure 1. **(b)** The whole transcriptome library construction method was simplified.

The gene specific library and the whole transcriptome library were constructed very differently (Figure 4). To identify the source of the bias, we carefully reviewed all steps at which library construction differed. Most importantly, the bias could have arisen from fragmentation or its following steps.

For the gene specific library, double stranded complementary DNA (dscDNA) fragmentation was performed with sonication, and adaptors were ligated to the fragmented dscDNA using DNA ligase (Figure 4a). Sonication and DNA ligase possibly introduced the extremely minor noise (Figure 3a and c). However, these two steps are not known to cause sequence bias, and reads in our experiment were not sufficiently biased to introduce read pile-ups and mapping gaps (Figure 2a).

By contrast, the whole transcriptome library construction method used RNaseIII fragmentation at the very beginning of the protocol (Figure 4b). Perhaps RNaseIII fragmentation causes a bias that has not been previously evaluated. However, the three steps following RNaseIII fragmentation (adaptor ligation, reverse transcription, and amplification) were already known to have sequence biases [4-6]. Therefore, we first inspected these three steps for potential problems.

We used RNA ligase for adaptor ligation. RNA ligase prefers to bind to certain phosphate donors, but preference patterns have been studied only for short oligomers [4]. In our case, phosphate donors were the fragmented single stranded RNAs (ssRNAs) of 200 base pairs (bp) in length. Figure 3a did not show the same sequence pattern as the short oligomer case [4]. Therefore, its sequence bias either appeared in a different way at the fragmented ssRNAs or was too small to be detected from whole transcriptome library data. We concluded that the sequence bias we saw was likely not caused by RNA ligase.

The bias for reverse transcription has been previously studied for the Illumina RNA-seq system [5]. The randomly primed reverse transcription of Illumina RNA-seq causes substantial sequence biases at the 5' and 3' ends. However, our reverse transcription method was different from the Illumina method, and their respective biases are likely different. While the random primers of Illumina RNA-seq can hybridize on any locations of mRNAs, the primers in our method are the overhangs of adaptors and can bind only to ends of fragmented ssRNAs. The overhangs are 4 bp random sequences, and they were used for reverse transcription. Because of this targeted priming, the sequence bias of the whole transcriptome library was entirely different from the Illumina random primer case (Figure 3a) [5]. Therefore, we concluded that it was unlikely that reverse transcription was a major source of bias in our library preparation.

Finally, amplification is known to have GC bias [6]. This bias could affect some transcript regions that might appear more or less highly expressed, but this could not generate 5' end sequence bias.

Therefore, we excluded the three steps adaptor ligation, reverse transcription, and amplification as major sources of 5′ end sequence bias of the whole transcriptome library. We next focused our efforts on RNaseIII. RNaseIII can digest ssRNAs of preribosomal RNA and bacteriophage T4 [13,14]. However, we removed ribosomal RNAs and used only human RNA samples. Even though we used only about 200 bp fragmented RNAs, their ssRNA digestion product length is different. During eukaryotic double stranded RNA (dsRNA) metabolism RNaseIII digests RNAs into 200 bp, and it is generally considered to be a random cutter. It recognizes double stranded RNA structures and cleaves them [14]. The RNA cleavage usually produces both short and long fragments. In yeast, the short fragments are about 28–32 bp in length and have unique sequence (AGNN) in the middle as containing hairpin structures [15]. In our system, the long fragments were around 200 bp and used for RNA-seq. The short fragments were too short for RNA-seq.

Because RNaseIII specifically recognizes RNA secondary structure and leaves a unique sequence in the short fragments, it is not likely to be a perfectly random cutter. Therefore, the long fragments could have an RNaseIII specific sequence pattern. On the basis of this reason, we replaced RNaseIII fragmentation by an alternative method using heat.

### Alternative library constructions

We fragmented RNA at 95°C when it was denatured. In denatured RNA the hydrogen bonds are broken and only covalent bonds remain. Denatured RNA has no secondary structure. Therefore, each base can be attacked equally by heat, and we do not expect that heat fragmentation can introduce 5′ end bias.

**Figure 5:**
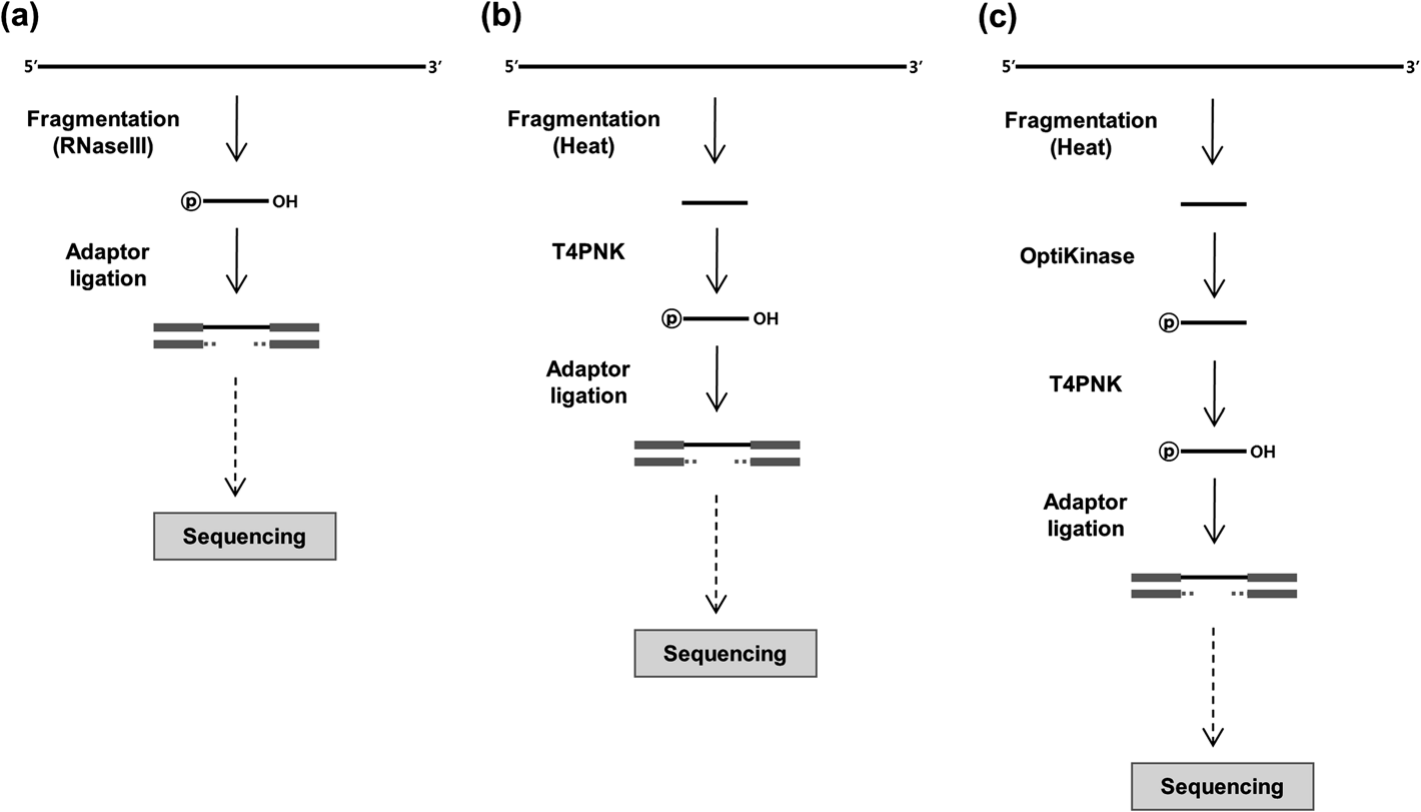
**Alternative fragmentation methods. (a)** RNaseIII was used for the standard fragmentation method. **(b)** Heat fragmentation method was used as an alternative fragmentation method. After heat fragmentation, T4PNK was applied to modify RNA fragment ends for adaptor ligation. T4PNK adds phosphate and leaves hydroxyl groups at their 5’ and 3’ ends, respectively. **(c)** Modified heat fragmentation method was designed using OptiKinase. OptiKinase reduces T4PNK bias introduced during the RNA fragment modification. Because OptiKinase has only kinase activity, T4PNK was used for phosphatase activity after OptiKinase treatment.

However, heat fragmented RNA could not be used directly for adaptor ligation. The 5' and 3' ends of the heat fragmented RNA needed to be modified. We used T4PNK for the modification (Figure 5b). Its kinase activity adds phosphate groups at 5′ fragment ends. Its phosphatase activity removes phosphates from at 3′ fragment ends and leaves hydroxyl groups.

T4PNK is known to have sequence bias in its kinase activity, according to the OptiKinase product information [16]. Therefore, we also tested an alternative preparation method where we applied OptiKinase before T4PNK treatment (Figure 5c). OptiKinase has been shown not to have the sequence bias of T4PNK, but it does not have phosphatase activity at the 3′ end. Here, we assumed that T4PNK has negligible bias in its phosphatase activity and used it following OptiKinase treatment for its phosphatase activity. (Our results confirmed that this assumption is valid, as shown below.) Note that for kinase activity, T4PNK treatment required the optimization for enzyme and substrate titration and treatment time [16]. Therefore, OptiKinase was clearly a better choice to avoid technical variation in RNA-seq studies.

In Figure 5, we compared the alternative whole transcriptome library construction methods with the standard whole transcriptome library construction. The standard method fragmented RNA using RNaseIII and ligated adaptors to the RNA (Figure 5a). For heat fragmentation method, we switched RNaseIII fragment to heat fragmentation and did adaptor ligation after T4PNK treatment (Figure 5b). Modified heat fragmentation method reduced known T4PNK bias of the heat fragmentation method as treating OptiKinase between fragmentation and T4PNK steps (Figure 5c).

To assess the sequence bias of these two newly proposed fragmentation methods, we prepared two new whole transcriptome libraries, together with a second iteration of the whole transcriptome library prepared according to the standard protocol based on RNaseIII (Figure 5a). To reduce batch-to-batch sequencing variation, we sequenced all three libraries simultaneously in the same SOLiD system. Their mapping and further data analyses were processed identically.

### Mapping results of novel libraries

**Figure 6:**
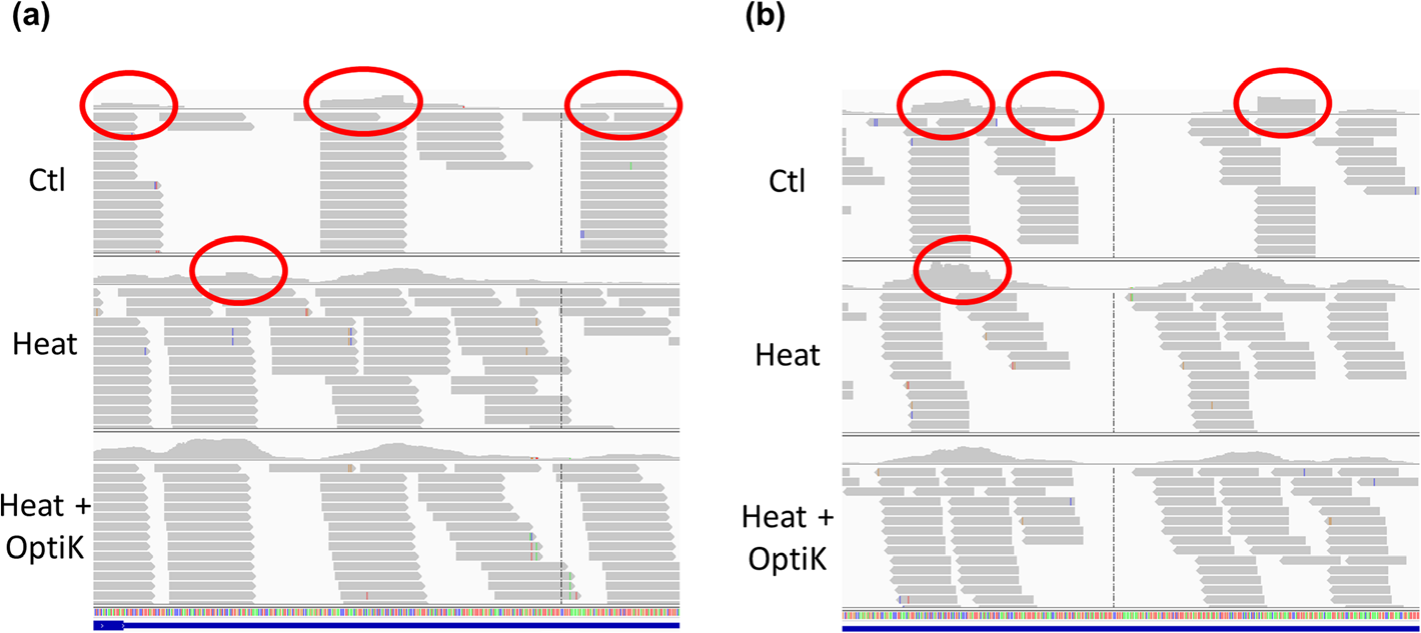
**Mapping patterns of the three whole transcriptome libraries. (a)** 5’ read mapping results of the three whole transcriptome libraries were visualized at IGV. Ctl refers to the library constructed using RNaseIII. Heat refers to the library constructed using the heat fragmentation method. Heat + OptiK refers to the library constructed using the modified heat fragmentation method. The pile-ups of duplicated reads are indicators for fragment end sequence bias. The red circles represent the pile-ups. Ctl showed pile-ups. Heat had fewer numbers of pile-ups, and no pile-ups were found at Heat + OptiK. **(b)** 3’ read mapping results show the same patterns as the 5’ read mapping.

We processed paired-end reads instead of previous single-end reads and visualized both 50 mer 5′ and 35 mer 3′ read mapping results (Figure 6). In the following, “Ctl” refers to the library prepared with RNaseIII, “Heat” refers to the library generated with heat fragmentation and T4PNK, and “Heat + OptiK” refers to the library that was generated with an additional OptiKinase step right after the heat fragmentation.

As described before for Figure 2 and 3, fragment end sequence bias generated the pile-up mapping pattern. Figure 6 compares the mapping patterns of Ctl, Heat, and Heat + OptiK for an endogenous control, PGK1 gene (Figure 6a). Ctl showed clear pile-ups for 5' read mapping results as expected. By contrast, Heat had much fewer pile-ups, and Heat + OptiK had virtually no pile-ups. Also, Heat + OptiK had the most even and smoothly connected distribution of the mapped reads.

Figure 6b shows mapping results for 3' reads. Ctl showed pile-up patterns just like in the 5' case. The pile-up patterns were weaker using Heat and largely disappeared using Heat + OptiK.

**Figure 7:**
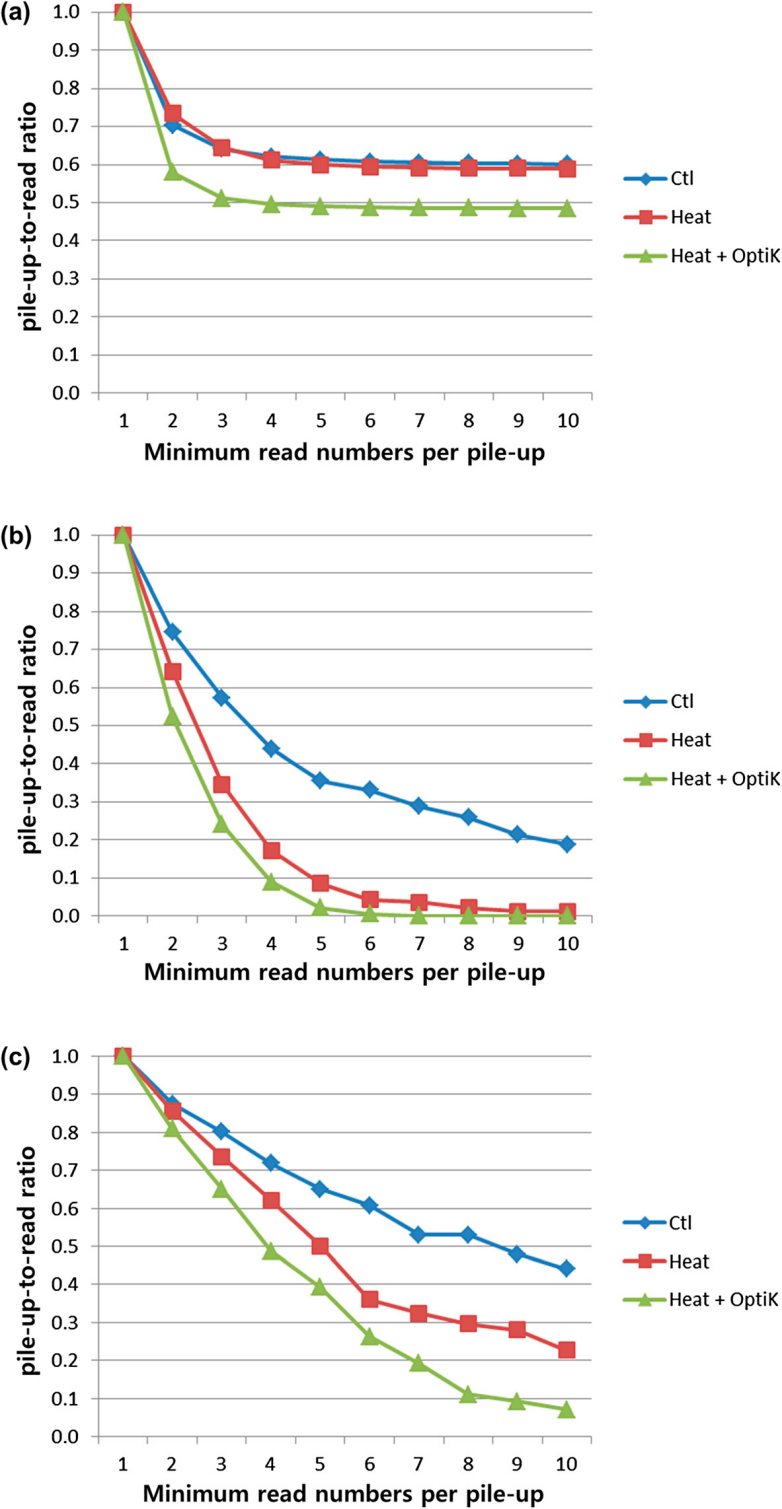
**Proportion of reads that contribute to pile-ups as a function of the minimum size of a pile-up.** Along Y-axes we have plotted the ratio between the number of reads mapping to pile-ups and the total number of all mapped reads (pile-up-to-read ratio). Along X-axes we have plotted the minimum number of reads that map to the same location to be considered a pile-up. The pile-up-to-read ratio is 1 when the minimum number of reads per pile-up is 1 since in this case all reads are considered to be part of a pile-up. As the minimum number of reads per pile-up is increased, the ratio declines. The magnitude of the decline is a measure of the extent to which the set of reads has a tendency to form pile-ups (faster decline means fewer pile-ups). **(a)** Genome-wide pile-up-to-read ratios of 5’ read mappings. **(b)** and **(c)** pile-up-to-read ratios for the GABAB1 gene and the ACTB gene, respectively. For both the genome-wide and the gene-specific cases, the pile-up-to-read ratios decrease in the order Ctl, Heat, and Heat + OptiK. Thus, Ctl has the highest tendency to form pile-ups, while Heat + OptiK has the lowest tendency.

To study genome-wide pile-up patterns using the Ctl, Heat, and Heat + OptiK libraries, we calculated pile-up-to-read ratios from the total mapped reads (Figure 7a). Genome-wide pile-up-to-read ratios for Heat are slightly smaller than for Ctl, but Heat + OptiK had a much lower ratio than the other libraries. The three libraries had similar total mapped reads numbers, about 5.3 million (Table S1). Therefore, the reason for the difference in genome-wide pile-up-to-read ratio is the library construction method.

For individual genes, the three libraries had different patterns of pile-up-to-read ratios (Figure 7b and c). For the GABAB1 gene, Heat and Heat + OptiK had much lower ratios than Ctl, and Heat + OptiK showed slightly smaller ratios than Heat (Figure 7b). For ACTB gene, the ratio of Heat was near the average of the Ctl and Heat + OptiK ratios (Figure 7c). Among these two genes, the ACTB gene is more highly expressed than the GABAB1 gene, and the pile-up-to-read ratios of the ATCB gene were higher than the ratios of the GABAB1 gene. Genes that have more mapped reads have more chances to have identical reads and higher pile-up-to-read ratios.

### Identifying the detailed sequence bias patterns of the three libraries Ctl, Heat, and Heat + OptiK

**Figure 8:**
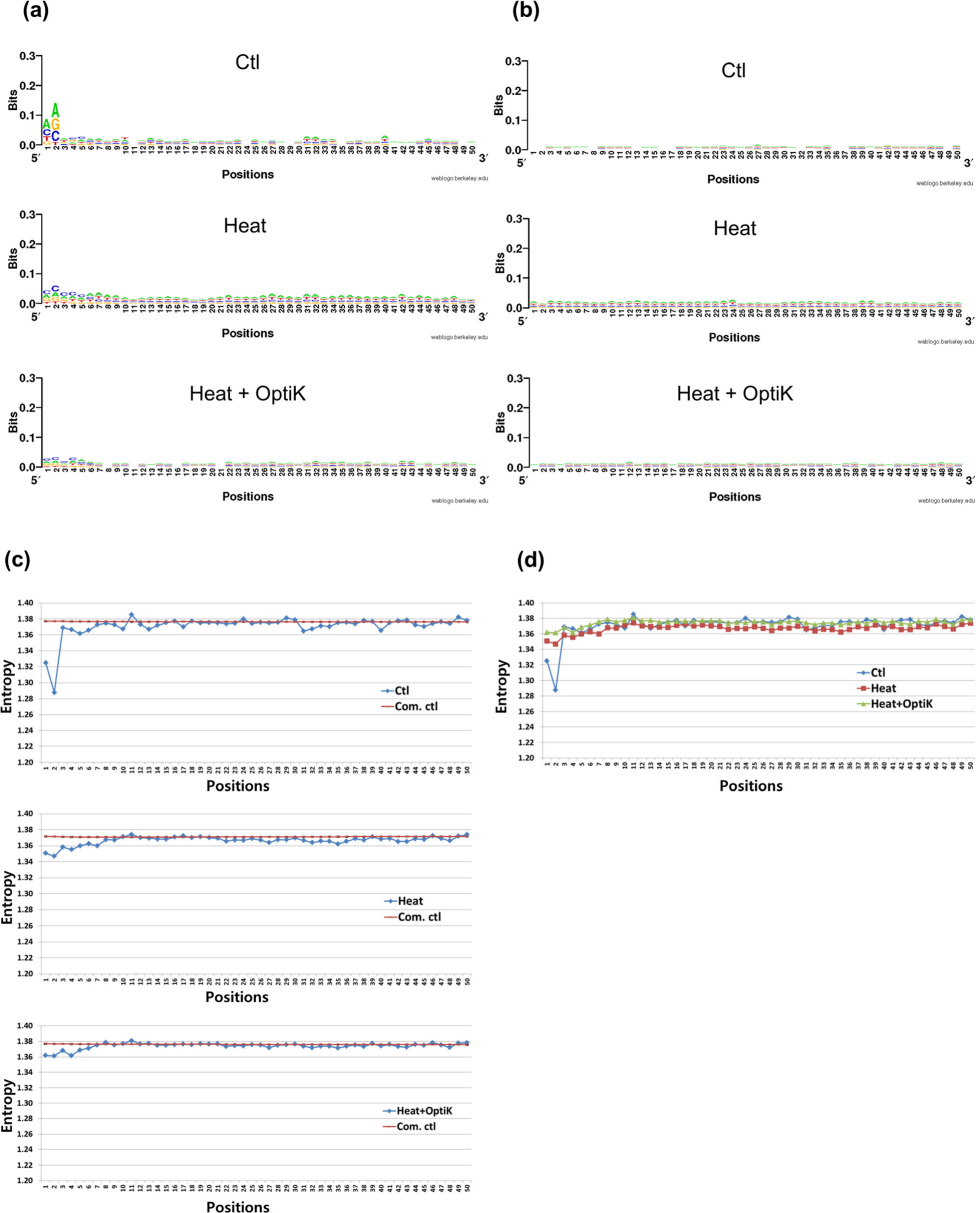
**Sequence logo and entropy analyses of mapped 5’reads. (a)** We compared sequence logo results for 10,000 randomly selected mapped 5’ reads of Ctl, Heat, and Heat + OptiK. **(b)** After generating computational control reads, sequence logo results were also compared. Ctl showed strong biases near the beginning of 5’ reads. Heat had less biases at 5’ but showed persistent low level biases at all nucleotide positions. The biases did not have the same sequence bias patterns as Ctl. Therefore, RNaseIII generates sequence biases at 5’. Though Heat + OptiK had a similar sequence bias pattern as Heat, it was much smaller throughout the entire nucleotide positions. Thus, OptiKinase weakens T4PNK sequence biases, and Heat + OptiK is the best method. **(c)** shows entropy results of the three libraries including the entropy data of their computational control reads (Com. ctl) for 5’ end reads. **(d)** The entropy results of the three libraries were compared. Lower entropy values correspond to greater sequence bias.

**Figure 9:**
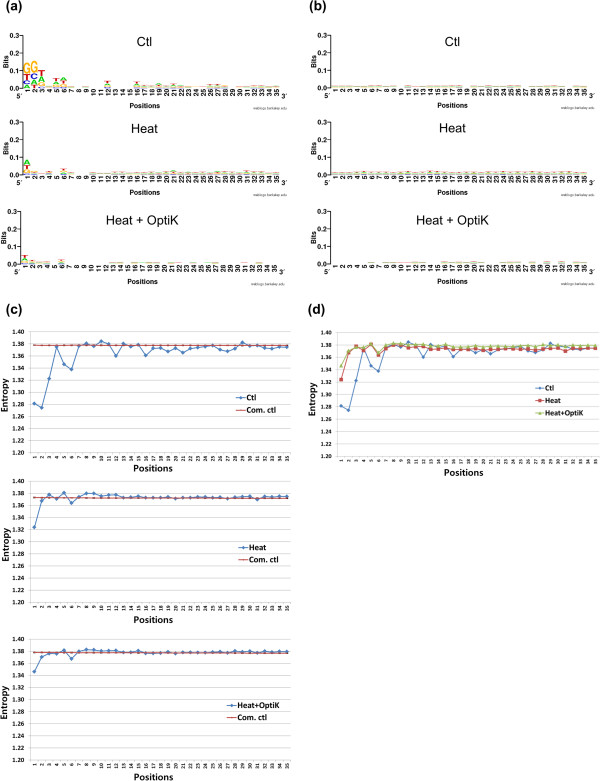
**Sequence logo and entropy analyses of mapped 3’reads. (a)** We also compared sequence logo data for mapped 3’ reads of Ctl, Heat, and Heat + OptiK. **(b)** After generating 35 bp computational control reads for the 3’ reads of the three libraries, sequence logo results were compared. Ctl also showed strong biases at the beginning of 3’ reads. Even though the biases of Heat were smaller, there were persistent low level biases at all nucleotide positions. Because the biases of Heat were not the same sequence bias patterns as Ctl, RNaseIII also generates sequence biases at 3’ ends. The 3’ end sequence bias, A or T, indicated the sequence bias of T4PNK phosphatase activity. Like 5’ reads, Heat + OptiK had a similar sequence bias pattern as Heat, but it was much smaller. Thus, OptiKinase weakens T4PNK sequence biases, and Heat + OptiK is also the best method for 3’ reads. **(c)** shows their entropy results including computational control data (Com. ctl). In **(d)** entropy data were compared.

Next, we carried out sequence logo and entropy analyses for the three libraries (Figure 8 and 9). Figure 8 represents results for 5' reads, and Figure 9 represents results for 3' reads. Figure 8b and 9b showed the sequence logo results for the computational control for Figure 8a and 9a. Computational controls were generated as for Figure 3, but for Figure 9 35 bp reads were prepared as controls for the 35 mer 3′ end reads.

As described before for Figure 3a, Ctl had a strong bias of AA at the 5′ end of reads and showed minor bias at the other positions (Figure 8a). Heat had less bias right at the 5′ end but had some bias throughout the entire 50 bp window. T4PNK is biased though its bias is somewhat less than the RNaseIII bias at the 5′ end. Finally, Heat + OptiK had almost no bias throughout the entire 50 bp window. Even though its negligible bias shares the same sequences with Heat, OptiKinase decreases the T4PNK sequence bias. For 5′ reads, Heat + OptiK was the least biased method overall.

For 3′ reads, biases of Ctl were generally more severe than for the 5′ reads (Figure 8 and 9), but the overall bias pattern followed that of the 5′ reads. Ctl showed the strongest bias at the beginning of reads with a preferred sequence of GG. Heat was biased towards sequences starting with A or T and had small bias ubiquitously throughout the sequence. The bias pattern was very different from the one found in Ctl. Thus, we believe that this bias was caused by the phosphatase activity of T4PNK. Most enzymes acting on nucleic acids are generally affected by nucleotide sequences near their reaction sites [4]. Surprisingly, Heat + OptiK showed a much smaller sequence bias for 3′ reads than Heat. Thus, it seems that pretreatment with OptiKinase weakens the bias of T4PNK phosphatase activity because the T4PNK mostly has phosphatase activity rather than kinase activity.

As expected, the entropy results generally mirrored the sequence logo results (Figure 8c and 9c). The entropy plots showed clearly that the entropy values for Heat + OptiK fell consistently below the entropy values for Ctl and Heat for both 5′ and 3′ end reads (Figure 8d and 9d). Therefore, Heat + OptiK is the least biased method for both 5′ and 3′ end reads.

As a result of our study, we have obtained the sequence biases of RNaseIII and T4PNK. T4PNK kinase activity has a minor bias at the 5′ end of fragmented ssRNA (Figure 8). T4PNK phosphatase activity is biased mostly towards sequences with an A or T nucleotide at the fragment end of 3′ reads (Figure 9). Its sequence biases make less severe pile-ups than the broad RNaseIII sequence biases (Figure 6b).

Even when the pile-up reads were filtered out from mapped reads of the initial whole transcriptome library and the Ctl library, the filtered reads still showed the same sequence bias patterns of RNaseIII with a smaller number of mapped reads (Additional file 3: Figure S2). Therefore, RNaseIII has a specific target sequence for its reaction.

**Figure 10:**
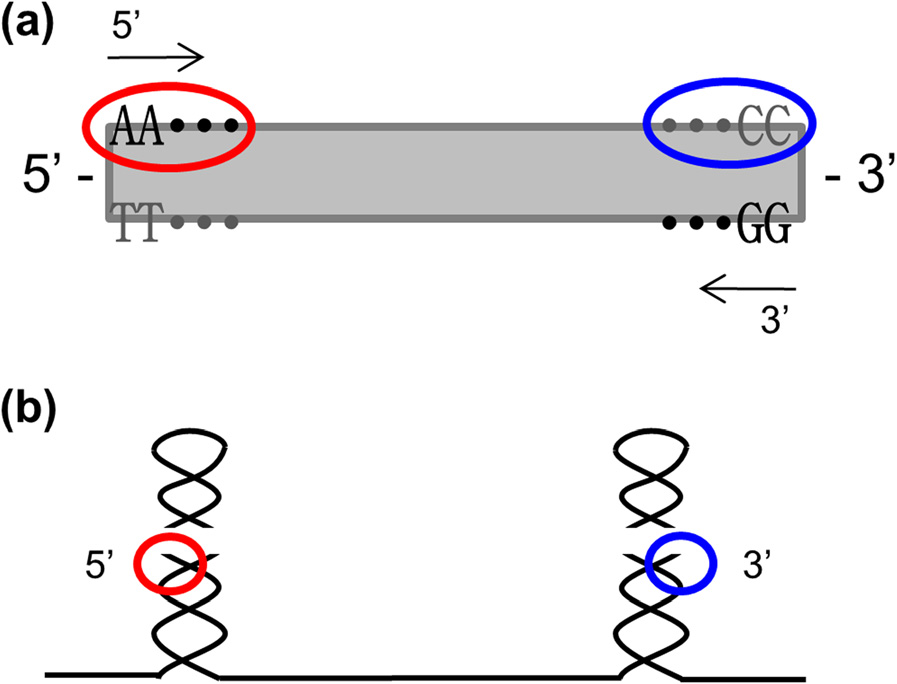
**RNaseIII specific cutting sites. (a)** Sequence biases of RNaseIII were summarized. RNaseIII specific cutting sites were mostly 5' - AA CC - 3'. (The 3' end reads are shown as reverse complement.) The red circle represents the 5' end cutting sites, and the blue circle represents the 3' end cutting site. **(b)** The figure shows how these cutting sites map onto the hairpin regions cut by RNaseIII. The sites are disconnected by short fragment RNAs. The RNaseIII specific cutting sites of our data are at the long fragment ends.

RNaseIII has biases at both the 5′ and 3′ ends (summarized in Figure 10a). Its sequence pattern was mostly 5′ - AA∙∙∙ ∙∙∙CC - 3′ (We reverse complemented the 3′ sequence here). Based on the known RNaseIII reaction mechanism, the two 5′ and 3′ biased sequences were separated by the short fragmented RNAs (Figure 10b). Thus, we have shown that RNaseIII introduces sequence biases into the long fragments that it produces.

## Discussion

Our RNA-seq data showed the power of next generation sequencing to find unexpected sequence biases from enzymes and to reveal previously unknown enzyme reaction sites. A large data set of RNA-seq reads allowed us to identify previously unknown sequence biases and targeting sites of two enzymes, RNaseIII and T4PNK.

RNaseIII was previously considered to be a random cutter for dsRNAs. However, our study has shown that it indeed has preferred cutting sites. RNaseIII recognizes RNA secondary structure and digests the middle of double stranded RNA [14,15]. Yeast RNaseIIIs recognizes the AGNN loop of the double stranded RNA [15]. Thus, it is not surprising that its cutting site is non-random even though it has never before been identified. Our high resolution study of RNA-seq found that RNaseIII caused pile-up mapping of RNA-seq data because of its specificity to certain digesting sites. As a result, RNaseIII yielded fragment ends with conserved sequences. Importantly, we have proposed novel RNA-seq library construction methods that solve the bias issue.

Similarly, whether T4PNK phosphatase activity had a sequence bias was not previously known even though the bias in its kinase activity was known for short oligomers. We found a bias in its phosphatase activity and a unique minor bias in its kinase activity for ssRNA fragments. The amount of bias in the library was strongly reduced when we treated RNA fragments with OptiKinase first. Apparently, when T4PNK is used for both kinase and phosphatase activities, its phosphatase activity is more strongly biased than when it is used for phosphatase activity only.

The pile-ups and gaps produced by the sequence biases cause erroneous results in the identification of SNPs and splicing junctions (which might be missed) and in the measurement of expression levels. Even though overall gene expression levels were probably not significantly affected by the pile-ups and gaps, a more precise expression analysis, e.g. a comparison of expression patterns among highly expressed short genes and exons, could be significantly affected by these biases.

RNA-seq based specific cutting sequence identification may provide useful applications in the prediction of miRNA genes. The proteins Drosha and Dicer belong to the RNaseIII family and produce miRNAs [17]. Similarly to what is shown for RNaseIII in Figure 10b, Drosha and Dicer also recognize hairpin structures. Drosha produces pre-miRNA primary transcript hairpin structures after digestion in the nucleus. Dicer detects the hairpin structures of the pre-miRNAs and carries out one more cleavage. While the cutting pattern of Dicer is partly understood [18], we believe that further insight could be obtained following an RNA-seq approach similar to what we carried out here for RNaseIII. An improved understanding of the cutting specificity of both Drosha and Dicer would then be useful for prediction of miRNA genes. Current prediction is mostly based on RNA secondary structure; mature miRNA sequences are primarily predicted by more than one hairpin precursor locus [19]. However, prediction accuracy tends to be low [17]. If specific cutting sites for these enzymes were known, this knowledge could be incorporated into methods of miRNA gene prediction.

## Conclusions

We have found that the standard RNA-seq protocol, SOLiD™ Total RNA-Seq Kit, has substantial sequence biases and pile-ups in mapped reads. The biases could be tracked to RNaseIII activity which preferentially cuts at specific locations. Other steps in library preparation, specifically RNA ligation, reverse transcription, and amplification, seemed to be free of bias in the RNA-seq preparation method. By changing fragmentation from RNaseIII to heat the sequence bias was largely eliminated, and the vast majority of the sequence pile-ups disappeared. Therefore, heat fragmentation was superior to commercial RNaseIII methods when preparing RNA-seq.

Among the various methods of library preparation we studied, Heat + OptiK was overall the least biased. However, while vastly improved over the manufacturer's protocol, it is not a completely unbiased method. Entropy calculations and sequence logos showed small amounts of bias near the beginning of both 5′ and 3′ reads. Some of the remaining biases at fragment ends seem to be caused by T4PNK. The origins of any other minor deviations from complete randomness are not clear. They may be due to any combination of the other steps in library preparation, i.e. RNA ligation, reverse transcription, PCR amplification, and so on. Unfortunately, it is impossible to exclude all of the possibly biased steps from RNA-seq library preparation due to current technical limitations. Therefore, the individual steps should be optimized or substituted for less biased steps.

## Methods

### Whole transcriptome library construction

Total RNA from human prefrontal cortex was obtained from Ambion (Austin, TX, USA). RNA quality was evaluated using the Agilent 2100 Bioanalyzer RNA Nano Chip (Agilent Technologies, Santa Clara, CA, USA). After treating samples with DNase (Ambion, Austin, TX, USA), rRNA was depleted from total RNA using the RiboMinus kit (Invitrogen, Carlsbad, CA, USA). rRNA depletion was verified by the Agilent 2100 Bioanalyzer RNA Nano Chip. Whole transcriptome libraries were prepared using the SOLiD™ Total RNA-Seq Kit (PN 4445374; Applied Biosystems, Carlsbad, CA, USA) according to the manufacturer’s protocol for SOLiD™ 4 System.

### Gene specific library construction

To prepare a gene specific library, gene specific primers for reverse transcription and amplification were designed at the 3′ UTR of human GABAB1. The primers were designed using Primer3 [20] and tested for binding specificity with PrimerSelect (DNASTAR, Inc., Madison, WI, USA). After treating the human prefrontal cortex total RNA with DNase, GABBR1-C-ter-GSP-2, 5′ - AGAGACACCACAGTGTGAAAGG - 3′, was used for reverse transcription with the SMARTer PCR cDNA Synthesis Kit (Clontech, Mountain View, CA, USA). Using a template switching method, dscDNA was generated [11]. For the amplification step, SMARTer2A-22mer, 5′ - AAGCAGTGGTATCAACGCAGAG - 3′, was designed based on the sequence of a provided primer, SMARTer2A Oligonucleotide, that was bound at the 5′ ends of transcripts during the template switching step. After amplification with SMARTer2A-22mer and GABBR1-C-ter-GSP-1, 5′ - AGGTCCATCTGTCTATCCCAAC - 3′, the dscDNA generation was confirmed with gel electrophoresis.

From the gel electrophoresis data, we optimized the number of amplification cycle based on band intensity. As soon as the band intensity reached maximum, the optimal amplification cycle was reached, and we chose that cycle number for further analysis. The chosen cycle had the lowest level of non-specific PCR products among the amplification cycles containing maximum levels of GABAB1 gene specific PCR products. The optimal amplification cycle was verified with quantitative real-time PCR experiments using TaqMan® Gene Expression Assays (Applied Biosystems, Carlsbad, CA, USA). Based on a previous GABAB1 study [12], the primer and probe set targeting the exon 22–23 junction of GABAB1 was used to measure gene specific amplification level. 18S and beta-glucuronidase (*GUSB*) were used for endogenous controls. For each amplification cycle, gene specific amplification levels were calculated from both endogenous controls. Among amplification cycles, a cycle was selected as soon as both gene specific amplification levels reached plateau. The selected cycle was the same as the optimal amplification cycle chosen from the gel electrophoresis data.

Gel extraction removed small amplification products and the primers for reverse transcription, template switching, and amplification. According to the SOLiD™ 3 System Library Preparation Guide (Applied Biosystems, Carlsbad, CA, USA), the remaining dscDNA was sonicated to about 200 bp length, and the gene specific library was prepared for SOLiD sequencing.

### Sequence mapping and visualization

The whole transcriptome and gene specific libraries were sequenced using the SOLiD system. For each library, TopHat was used for sequence mapping against hg18 [1]. During mapping, reads that had low quality values were difficult to map and increased total mapping time. To eliminate these issues, we set up quality filter parameters to remove the reads. For every read, we counted the number of quality values that were below 8 (higher than 15% probability of incorrect base call). If the numbers were larger than 13 at 50 mer sequence reads, the reads were filtered out. For 35 mer sequence reads, reads were removed if the numbers were over 9. Non-coding RNAs (mostly rRNA and tRNA) and adaptors (including gene specific primers) were filtered out using the Mapreads mapper (Applied Biosystems, Carlsbad, CA, USA). Two mismatches out of 20 mer reads were allowed for the non-coding RNAs and adaptors mappings. For TopHat mapping, the remaining reads were also converted to .fq files using fq_all2std.pl [21]. To visualize the mapping data, .sam files were processed by igvtools [22] and visualized with the Integrative Genomics Viewer (IGV) [23].

### Sequence analysis

A fraction of the reads we obtained mapped uniquely to a single location in the genome while another fraction mapped to multiple locations. To include the reads that mapped to multiple locations in our analysis without artificially inflating expression counts, we assigned each multiple mapped read to exactly one mapped location, chosen at random. Whether we included or excluded multiple mapped reads did not qualitatively alter the conclusions we could draw from our experiments (data not shown).

From all the mapped reads, we calculated entropy ($H=-\sum_{i}^{A,T,G,C}P_{i}\log P_{i}$) at each nucleotide position. Here, *P*_A_, *P*_T_, *P*_G_, and *P*_C_ represent the relative abundances of each of the 4 bases (A, T, G, and C). Among them, a random sample of 10,000 reads was used as input for sequence logo (a graphical representation of a conserved sequence pattern) [24,25]. For the gene specific library, we removed reads that contained the primer sequences used for amplification, and the remaining mapped reads were used for entropy calculation and sequence logo.

As computational controls, for each library we computationally generated random exome sequences. Based on mapped read locations and splicing junction data, we predicted a possibly existing 200 bp cDNA sequence in the RNA-seq library for each mapped read. We randomly took continuous 50 bp or 35 bp sequences from the individual 200 bp cDNA sequence. For entropy calculations, we generated these control reads 1000 times. However, WebLogo cannot take multiple sequence data inputs to generate a single sequence logo. Therefore, we generated random sequences without iteration for the sequence logo study.

### Heat fragmentation library construction

We evaluated two alternative heat fragmentation methods. Under the first method, 300 ng rRNA depleted total RNA was added into nuclease free water (Ambion, Austin, TX, USA) up to 4.5 ul. Using a PCR machine, the RNA containing 0.2 ml tube was incubated at 95°C for 80 minutes. Fragmented RNAs were verified using the Agilent 2100 Bioanalyzer RNA Nano Chip. The size of RNA fragments was around 200bp. For the second method, we performed heat fragmentation in buffers containing divalent ions following a previous study [11] and other on-line protocols [26,27]. Their incubation time was shorter (10 minutes) than in the nuclease free water. Agilent 2100 Bioanalyzer RNA Nano Chip data verified that the second fragmentation method produced comparable RNA fragment size to the ones produced by the first fragmentation method. The incubation time for both methods was optimized based on PCR machines, incubation temperature, solutions, RNA sources, and tube shapes.

After heat fragmentation, RNA ends had to be modified to allow adaptor ligation. We used T4PNK (USB, Cleveland, OH, USA) to add phosphate groups to 5′ ends and leave hydroxyl groups to 3′ ends. Adaptor ligation and subsequent steps of library construction were identical to the manufacturer′s protocol of the SOLiD™ Total RNA-Seq Kit (Applied Biosystems, Carlsbad, CA, USA).

### Updated heat fragmentation library construction

To minimize known sequence bias of T4PNK, we introduced an additional treatment step to the heat fragmentation library construction. After heat fragmentation, we used OptiKinase (USB, Cleveland, OH, USA) to add phosphate groups to the 5′ ends of fragmented RNAs. Subsequently, we proceeded with T4PNK as before to leave hydroxyl groups to the 3′ ends of fragmented RNAs.

For all library constructions except gene specific library construction, we used the same PCR cycle for amplification to minimize amplification artifact variation. We prepared mostly two more replicate libraries to confirm the sequence bias patterns. For all whole transcriptome and gene specific libraries, we did the same sequence mapping and visualization.

## Reviewers' comments

### Reviewer's report 1: Dr. A. Kolodziejczyk (nominated by Dr. Sarah Teichmann, MRC Laboratory of Molecular Biology, United Kingdom)

The existence of biases caused by preparation of sequencing libraries is well known. In previous studies, several issues were investigated, such as GC content or length bias due to PCR amplification. This paper shows that RNase III is a source of 3’ and 5’ bias, which was not known before and is complementary to previous research.

In the paper the authors show a comparison of a gene-specific library constructed using primers specific for the gene’s 3’ UTR and a whole transcriptome library. One can see that the gene specific library has significantly less ‘pile ups’. The authors suggest that the reason may be read duplication under SOLID sequencing due to 5’ bias in the transcript. As the libraries were constructed using very different methods, one cannot conclude the precise causes of the differences between them.

In the results section, it is mentioned that different primers were used for construction of the libraries, but the fact that authors used completely different protocols to create them is omitted. As stated in the paper, the method of library preparation is crucial for any biases that may occur, so the authors should make it clearer that substantially different methods were used to create these libraries.

**Author’s response:** Thank you for pointing this out. We have revised the first section of Results so that it now clearly spells out the substantial difference between the preparation methods used for the gene specific library and the whole transcriptome libraries.

It would also be informative to compare not only how reads map to GABAB1 using gene-specific and whole transcriptome libraries, but also libraries done using improved protocols.

**Author’s response:** We have expanded Results and Methods sections to include this information.

Subsequent experiments show that biases introduced by enzymes cause pile ups. The authors systematically test enzymes used in library preparation for 3’ and 5’ biases. They identify the cause of these biases and show alternative method to minimize them in a very straightforward and clear way.

This discovery will help scientists to construct sequencing libraries for the SOLID system. It may also be used to improve methods for computational bias correction in the data. Additionally it also gives a basic answer about the sequence preference of RNase III.

In the discussion it is suggested that as Dicer and Drosha are RNase III family enzymes, the knowledge of sequence preference of RNase III will help to improve miRNA prediction accuracy. Since these proteins do not belong to the same subgroup within RNase III family, it is very unlikely that they share the same cutting site. The recognition sequences of Drosha and Dicer have been studied, and it was shown that it is the 5’ end of pre-miRNA that is recognised by Dicer [18].

**Author’s response:** We appreciate these comments. We have revised the Discussion accordingly, and we now emphasize that a study similar to ours could be performed to improve our understanding of the sequence specificity of Drosha and Dicer.

To sum up, this paper shows a good set of experiments that in thorough way test the effect of enzymes used in library preparation on the 5’ and 3’ biases.

### Reviewer's report 2: Dr. Eugene Koonin, NCBI, NLM, NIH, United States of America

This manuscript addresses a methodological problem of great importance, namely the sequence biases of the RNA-seq method. In the Introduction, the authors note that "For example, one of the next generation sequencing techniques, RNA-seq, may ultimately replace microarrays" which I think is an understatement. The RNA-seq method has already superseded microarrays for the study of gene expression and is extremely widely used of other purposes as well. Thus, the issue of biases looms large. The results presented here are potentially important as the authors detect inherent biases associated with RNAse III cleavage and the show that the extremely simple method of heat fragmentation (combined with T4 kinase treatment) alleviates the bias.

**Author’s response:** We have revised the statement on microarrays to better reflect the current state of the field.

I do not have any problems with the technical aspects of the article although I have to admit that I am familiar with RNA-seq only at the level of data analysis not data generation. What I am somewhat unhappy with, is the following final sentence of the RESULTS section of the abstract: "The identification of RNaseIII target sequences could improve microRNA (miRNA) gene prediction accuracy if other members of the RNaseIII family, including Drosha and Dicer, also have specific target sequences". And then, the final sentence of the CONCLUSIONS, also in the abstract: "This is the first application of RNA-seq to discover unknown enzyme target sequences." This theme does not show again until the Conclusions in the main text, and as such, these statements in the abstract, however interesting in principle, seem a sort of non-sequitur. The if in the first of the quoted sentences, regarding the specificity of Dicer and Drosha is a huge IF, and the claimed application of RNA-seq to target identification is not really developed in the article. In my view, it would be much better to either develop these ideas more carefully, at least in the Discussion, or drop them altogether. The article is useful and potentially important without this excursion into a somewhat different area.

**Author’s response:** We agree with this concern. We have removed this topic from the Abstract, and we have modified the Discussion as explained in our response to Dr. Kolodziejczyk.

### Reviewer's report 3: Dr. Christoph Adami, Michigan State University, United States of America

This reviewer provided no comments for publication.

## Abbreviations

SNP: Single nucleotide polymorphism; UTR: Untranslated region; cDNA: Complementary DNA; rRNA: Ribosomal RNA; tRNA: Transfer RNA; dscDNA: Double stranded complementary DNA; ssRNA: Single stranded RNA; bp: Base pairs; T4PNK: T4 Polynucleotide Kinase; miRNA: microRNA; ORF: Open reading frame; sscDNA: Single stranded complementary DNA.

## Competing interests

The authors declare that they have no competing interests related to this paper.

## Authors’ contributions

CL conceived research and designed experiments; CL and JKW conducted experiments; CL and COW designed computational and analytical methods; CL analyzed data; CL drafted the manuscript; CL, COW, RAH, and RDM contributed to final manuscript writing and its revision; All authors read and approved the final manuscript for publication.

## Supplementary Material

Additional file 1: Table S1 Mapping statistics of RNA-seq libraries. Click here for file

Additional file 2: Figure S1 Read pile-ups and gaps of the whole transcriptome library mapping. The pile-up mapping patterns and gaps were identified for several genes (typical housekeeping genes), ACTB, PPIA, GAPDH, and PGK1. They are shown in (a), (b), (c), and (d), respectively. Click here for file

Additional file 3: Figure S2 Sequence bias pattern without pile-up reads. After removing pile-up reads from mapped reads of whole transcriptome library and Ctl, their sequence bias patterns of RNaseIII were assessed. (a) Sequence logo and entropy were calculated after filtering out pile-up reads from whole transcriptome library mapped reads. (b) and (c) For Ctl we also did sequence logo and entropy analysis without pile-up reads. (b) is for 5’ reads, and (c) represents 3’ reads. Click here for file
