# Supplementary figures and images for: RNaseIII and T4 Polynucleotide Kinase sequence biases and solutions during RNA-seq library construction
Source: Biol Direct. 2013 Jul 4;8:16. doi: 10.1186/1745-6150-8-16 (PMC3710281; doi:10.1186/1745-6150-8-16)

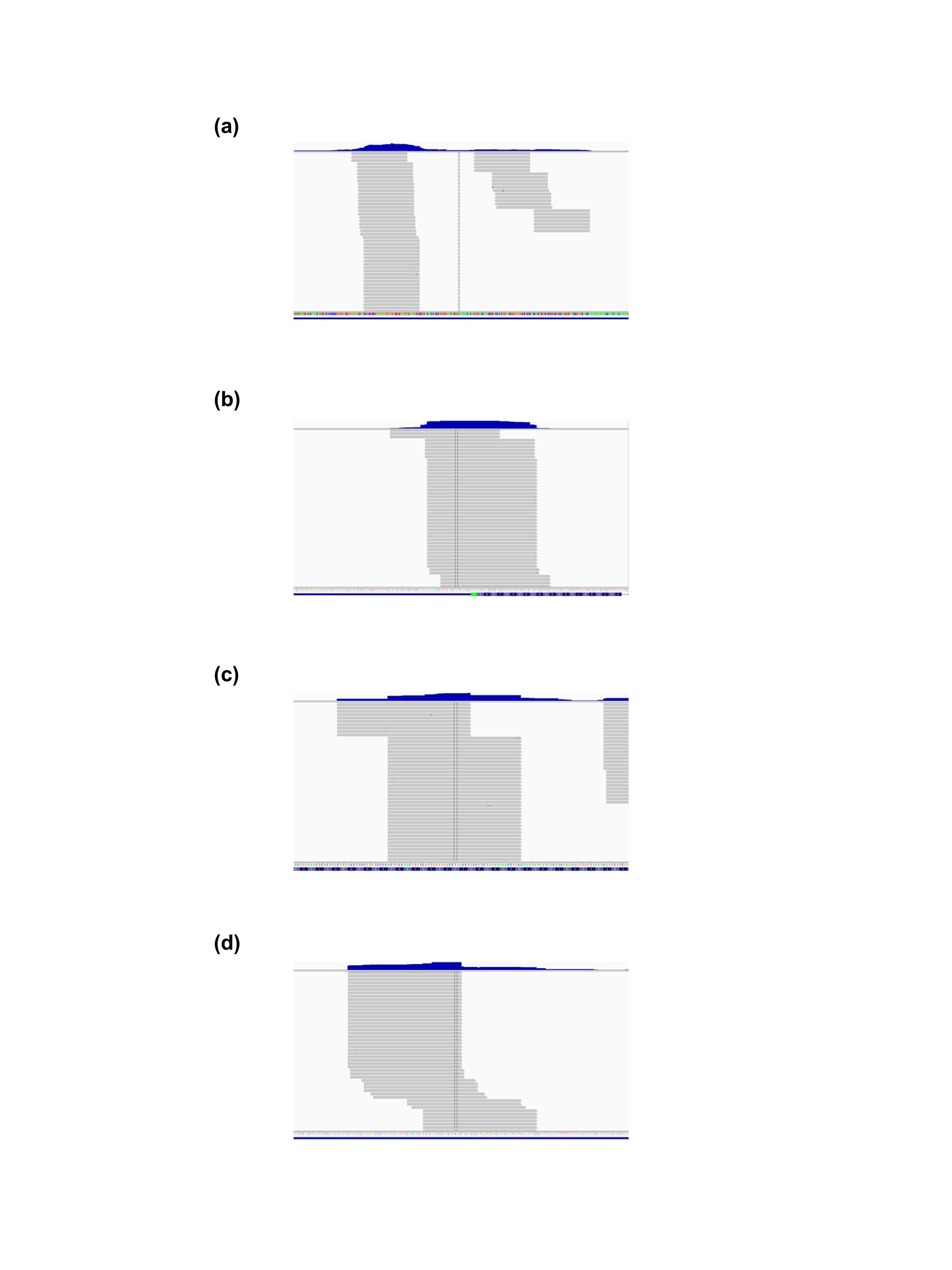

Supplement: Additional file 2: Figure S1 — Read pile-ups and gaps of the whole transcriptome library mapping. The pile-up mapping patterns and gaps were identified for several genes (typical housekeeping genes), ACTB, PPIA, GAPDH, and PGK1. They are shown in (a), (b), (c), and (d), respectively. [file 1745-6150-8-16-S2.tiff]

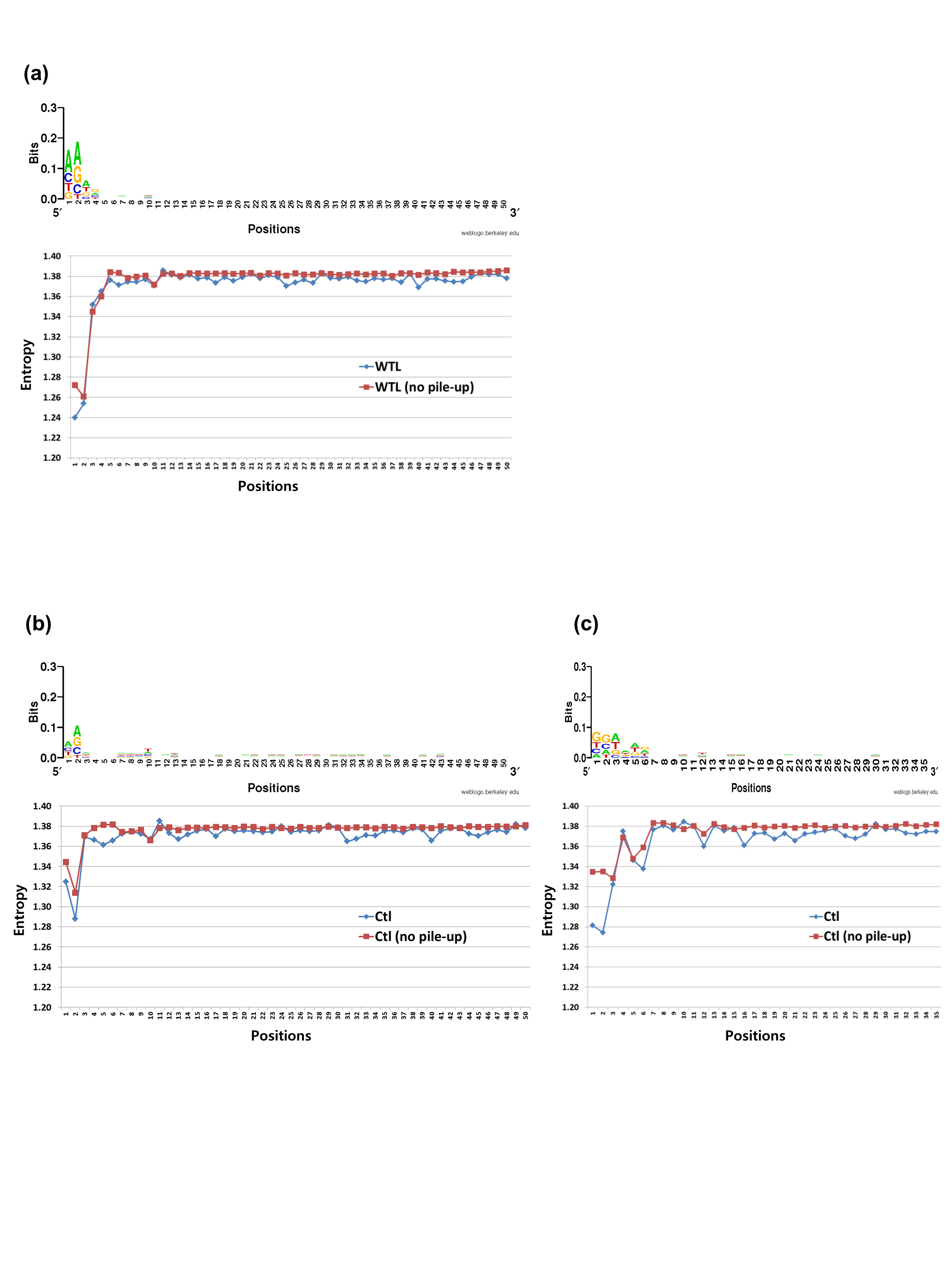

Supplement: Additional file 3: Figure S2 — Sequence bias pattern without pile-up reads. After removing pile-up reads from mapped reads of whole transcriptome library and Ctl, their sequence bias patterns of RNaseIII were assessed. (a) Sequence logo and entropy were calculated after filtering out pile-up reads from whole transcriptome library mapped reads. (b) and (c) For Ctl we also did sequence logo and entropy analysis without pile-up reads. (b) is for 5’ reads, and (c) represents 3’ reads. [file 1745-6150-8-16-S3.tiff]
